# Activating KRAS Mutations Expressed in 3D Endothelial Spheroids Induce Blebbing Morphologies Associated with Amoeboid-like Migration

**DOI:** 10.3390/cells15010022

**Published:** 2025-12-22

**Authors:** Lucinda S. McRobb, Vivienne S. Lee, Marcus A. Stoodley

**Affiliations:** Macquarie Medical School, Faculty of Medicine, Health, and Human Sciences, Macquarie University, Sydney, NSW 2109, Australiamarcus.stoodley@mq.edu.au (M.A.S.)

**Keywords:** adeno-associated virus, arteriovenous malformation, endothelial cells, KRAS oncogene: spheroid

## Abstract

**Highlights:**

**What are the main findings?**

- Endothelial spheroids expressing KRAS^G12V^ exhibit characteristic features associated with abnormal vessel development in arteriovenous malformations as well as novel phenotypes not previously observed in 2D monolayers.
- Expression of KRAS^G12V^ can induce blebbing morphologies associated with mesenchymal-to-amoeboid transitions (MAT) and amoeboid-like migration in brain endothelial spheroids after extended culture.

**What are the implications of the main findings?**

- Amoeboid migration may play a role in the aberrant angiogenesis of KRAS-driven arteriovenous malformations or in resistance to inhibitors targeting mesenchymal migration alone.
- 3D brain endothelial spheroids transduced with adeno-associated viral constructs offer a novel platform to investigate the plasticity of driver mutations associated with arteriovenous malformations.

**Abstract:**

Introduction: A 3D endothelial spheroid model expressing mosaic gain-of-function KRAS mutations was established to further understand the molecular changes associated with sporadic brain arteriovenous malformations (AVMs). Methods: Repellent 96-well U-bottom plates were seeded with human cerebral microvascular endothelial cells and resultant spheroids transduced with recombinant adeno-associated virus expressing KRAS^G12V^. Spheroids were monitored using live-cell imaging for extended culture periods. Results: In the early growth period, KRAS^G12V^ expression increased spheroid growth rates and enhanced spheroid sprouting on gel matrices consistent with known AVM characteristics. With extended culture, novel endothelial characteristics were observed. KRAS^G12V^-expressing spheroids displayed dynamic blebbing associated with the formation of rounded, hypertrophic cells disposed to engage in spheroid escape. These cells displayed reduced cell–cell adherence with rapid plasma membrane blebbing characteristic of amoeboid-like migration and mesenchymal-to-amoeboid transition. Spheroid growth and blebbing were reversed with MEK and mTOR inhibitors; Rho/ROCK inhibition specifically targeted the blebbing phenotype. Conclusions: Endothelial spheroids expressing KRAS^G12V^ exhibit characteristic features associated with abnormal vessel development in brain AVMs as well as novel phenotypes not previously observed in 2D monolayers. The ability to extend culture periods in this simple 3D model may allow further phenotypic exploration of important AVM driver mutations.

## 1. Introduction

Brain arteriovenous malformations (AVMs) are focal vascular deformities where direct artery to vein connections increase the risk of rupture and hemorrhagic stroke [1,2]. Approximately 60–70% of sporadic AVMs associate with post-zygotic acquisition of mutations in the Kirsten rat sarcoma viral oncogene homolog (KRAS) [3,4,5,6,7,8]. These activating, gain-of-function mutations are restricted to the AVM endothelium and are mosaic in their distribution [3]. Mutations commonly reside at glycine 12 of the KRAS protein with aspartate (G12D) and valine (G12V) substitutions predominating. Reconstitution of these mutations in zebrafish and mice has confirmed their role as independent drivers of AVM-like vascular malformations and as therapeutic targets [4,9,10,11,12]. Inhibitors targeting signaling pathways downstream of KRAS have been employed to reverse KRAS-associated phenotypes in cell and animal models [3,4,9,10,12] and in a limited capacity in humans [13].

Repurposing approved drugs for the treatment of AVMs may fill a therapeutic void for patients currently untreatable by traditional surgical or focussed radiation approaches. In vitro models can provide methods of rapid drug assessment in the discovery phase. Simple 3D endothelial spheroids may represent an ideal first-pass model to study the molecular and phenotypic changes associated with KRAS mutation and may bridge the gap between 2D and in vivo environments for early drug assessment. The physiological relevance of 3D culture over 2D monolayers has not been well established for endothelial cells, however early studies demonstrated increased endothelial maturation and survival in response to cell-to-cell adherence [14]. The ability of 3D endothelial spheroids to remain viable and stable for extended periods of study relative to 2D cultures may yet provide additional knowledge regarding the effects of KRAS mutations on endothelial behavior, to better understand KRAS-associated AVM pathophysiology, and to potentially fast-track the assessment of inhibitory drugs or the detection of KRAS-associated biomarkers for diagnostics or for targeted drug delivery.

In this study, we employ our recently established spheroid platform, that uses simple adeno-associated viral (AAV) transduction of brain microvascular endothelial cells for extended, mosaic mutant KRAS expression [15]. We examine the morphological and phenotypic effects of KRAS^G12V^, one of the most common gain-of-function mutations identified in brain AVMs. We demonstrate that this model displays characteristic angiogenic properties of AVM blood vessels and describe novel morphological features that may further our understanding of KRAS-mediated pathology and plasticity in the endothelium.

## 2. Materials and Methods

### 2.1. Cell Culture and Spheroid Formation

Immortalized human cerebral microvascular endothelial cells, hCMEC/D3 (CELLutions Biosystems Inc., Toronto, ON, Canada), were cultured in EBM-2 medium (Lonza, Basel, Switzerland) supplemented with 5% fetal bovine serum, 1% penicillin/streptomycin, 10 mM HEPES (Life Technologies, Grand Island, NY, USA), and 1 ng/mL human basic fibroblast growth factor (Sigma-Aldrich, North Ryde, Australia) [15,16,17]. All cells were grown at 37 °C in 5% carbon dioxide and were passaged at 90% confluence with 0.1% trypsin-EDTA (Life Technologies). U-bottom, 96-well plates with a non-adherent surface (Nunclon Sphera 96-well, Nunclon Sphera-Treated, U-Shaped-Bottom Microplate, #179425, Thermofisher Scientific, Waltham, MA, USA) were used for spheroid formation using cells seeded at 1000 cells/well [15]. Under these conditions, cells spontaneously aggregated and formed stable spheroids within 3 days. Immortalized HUVEC-TERT2 (CRL-4053TM, ATCC; Lonza, Basel, Switzerland) were sourced and grown as previously described [15]. The latter cells were only used for early transduction experiments and Western blotting.

### 2.2. AAV Construction and Transduction

All recombinant AAV constructs, and virus suspensions, were created and supplied by VectorBuilder (Chicago, IL, USA). All plasmid constructs were packaged into AAV2^QUADYF^ virus particles [18]. A mammalian gene expression vector was used to drive gene expression from a CMV promoter. Three constructs were used in this study: AAV-GFP (Vector name: pAAV[Exp]-CMV > EGFP:WPRE; VectorBuilder ID VB010000-9394npt) encoded green fluorescent protein and was used as a control to establish mosaic gene expression as previously described [15]; AAV-STUFFER (Vector name: pAAV[Exp]-CMV > STUFFER:WPRE; VectorBuilder ID VB240124-1165qxa) encoded a non-specific bacterial gene for use as a negative AAV control; AAV-mKRAS expressed the human KRAS gene with a valine substitution at glycine 12 (Vector name: pAAV[Exp]-CMV > (hKRAS[NM_004985.5](G12V)):WPRE; VectorBuilder ID VB230717-1044hnx). Stock solutions (10^11^–10^12^ GC/mL) were diluted in phosphate-buffered saline (PBS) before addition of 10 µL to each appropriate well (MOI of 100,000). Viral particles were added to full growth medium and could be left for extended culture periods without observations of cell death or toxicity [15]. For all experiments, media was not supplemented or exchanged at any time over the course of each experiment to prevent exogenous growth factor stimulation.

### 2.3. Western Blotting

To assess KRAS expression after AAV transduction, total protein was extracted after 5 days and 20–40 µg separated by SDS-PAGE and transferred to PVDF membranes as previously described [19]. Membranes were probed with an antibody specifically recognizing G to V substitutions in RAS proteins (rabbit polyclonal, #144125, Cell Signalling Technology Inc., Danvers, MA, USA)**,** or Total RAS (rabbit polyclonal, #3339T, Cell Signalling Technology Inc.) as well as phosphorylated ERK1/2 (#9101, Cell Signalling Technology Inc.) and species-specific secondary antibody linked to horseradish peroxidase (HRP) (goat anti-rabbit IgG, #ab6721, Abcam, Cambridge, UK). Bands were detected using enhanced chemiluminescence and quantitated using densitometry (NIH Image J v1.54f) [20].

### 2.4. Live-Cell Imaging

An Incucyte SX5 Live-Cell Analysis System (Sartorius, Goettingen, Germany) was used to digitally monitor spheroids in 96-well round-bottom plates and measure spheroid formation (area, size) using bright field or fluorescent settings (ex461 nm, em524 nm) to assess autofluorescence, FITC, or eGFP expression in real-time, as described previously [15]. Raw data and time-lapsed videos were created with Incucyte Base Analysis Software (v2023A Rev2) and exported for further analysis.

### 2.5. Immunocytochemistry and Microscopy

Immunocytochemistry was performed on fixed hCMEC/D3 spheroids in the U-bottom, non-adherent plates, as previously described [15]. Antibodies against CD31/PECAM-1 (mouse monoclonal, sc-376764, Santa Cruz, Dallas, TX, USA), or RAS^G12V^ (rabbit polyclonal, GTX132694, Genetex, Irvine, CA, USA), were used with species-specific AlexaFluor647-conjugated secondary antibodies (Life Technologies, Grand Island, NY, USA). Nuclei were counterstained with 4′,6-diamidino-2-phenylindole dihydrochloride (DAPI, 5 μg/mL, Life Technologies). Two-dimensional images were taken under fixed parameters using a Zeiss LSM800 Confocal Microscope (Jena, Germany) or EVOS FL Digital Inverted Microscope equipped with Phase, Blue (DAPI), Red (Texas RED), and Green (FITC) filter sets (Invitrogen, Waltham, MA, USA, AMF4300, AMEFC4300) [15].

### 2.6. Viability and Cell Death Assays

FITC-labeled annexin V (ANV-FITC) (1/100 dilution, A13199, Invitrogen) and propidium iodide (PI) (final concentration of 0.25 µg/mL, #556463, BD Pharmingen, Franklin Lakes, NJ, USA) were added 4h prior to imaging as markers of early apoptotic and late apoptotic cells, respectively. Calcein AM Blue Viability Dye (65-0855-9, Invitrogen) was dissolved in dimethylsulfoxide (DMSO), added at a final concentration of 10 µg/mL, and imaged 15–30 min after addition using the EVOS FL Digital Inverted Microscope.

### 2.7. Spheroid Sprouting Assays

Spheroids were grown for 2 weeks after transduction before transfer to flat-bottom 96-well plates and embedding in Geltrex (LDEV-Free Reduced Growth Factor Basement Membrane Matrix, Thermofisher) to assess sprout formation. Briefly, 50 µL of Geltrex was layered on the bottom of each well and set for 30 min at 37 °C. Spheroids were then transferred in 50 µL of the existing growth medium to the Geltrex layer; fresh medium was not added to prevent exogenous growth factor stimulation. A top layer of 70 µL Geltrex was added to the well and allowed to set for a further 30 min. Phase contrast images were taken at time zero and at 12–24h intervals on the EVOS FL Digital Inverted Microscope. Sprout number was counted manually after image conversion using the ‘Find Edges’ process in NIH Image J v1.54f [20].

### 2.8. Spheroid Dispersion and Cell Size Estimation

Spheroids were digested with trypsin 2–3 weeks after AAV transduction to examine changes in cell size. All steps were performed in the 96-well Nunclon Sphera plates. Spheroids were washed 3 times by PBS exchange before addition of 0.1% trypsin-EDTA. Spheroids were incubated at 37 °C for up to 30 min with occasional pipetting to expedite disruption. After complete spheroid dispersion (noting that acellular spheroid cores remained intact), cells were allowed to settle at the bottom of the round-bottom wells before phase contrast imaging with the EVOS FL Digital Inverted Microscope. Cell diameters were measured and averaged using scaled images in NIH Image J v1.54f [20].

### 2.9. Pathway Inhibition

**Figure 1:**
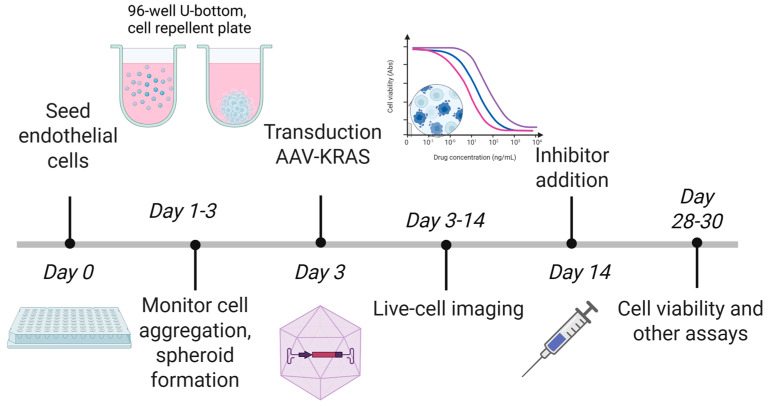
Overview of steps in 3D spheroid formation and culture used in this study. Created with BioRender.com on 10 December 2025 by McRobb, L., https://BioRender.com/nqlec51.

Stock solutions of MEK inhibitor (UO126, #662005, Merck, Darmstadt, Germany), mTOR inhibitor (Rapamycin, #11038, Cayman Chemical, Ann Arbor, MI, USA) and Rho/ROCK inhibitor (Y27632, #688000, Merck) were prepared in DMSO before dilution in PBS, drugs were added to each well in a total volume of 10 µL to a final concentration of 10 µM. Spheroids were grown for two weeks after AAV transduction before drug treatment. Spheroid morphology was monitored by live-cell imaging for 2–3 weeks after drug treatment. No media, serum, or growth factor replacement occurred during the course of each extended spheroid experiment. An overview of the steps of spheroid creation, spheroid monitoring, and inhibitor addition is shown in Figure 1.

### 2.10. Statistical Analysis

Prism version 9.3.0 (GraphPad, La Jolla, CA, USA) was used to graph data and generate statistical analyses. All data are shown as mean ± standard deviation (SD) for individual experiments with intra-assay replicates and mean ± standard error measurement (SEM) for inter-assay data representative of at least 3 independent experiments. Simple linear regression was used to compare spheroid growth rates. For parametric analysis, one-way ANOVA was used with Tukey’s post hoc analysis. For non-parametric analysis, Kruskal–Wallis was used with Dunn’s post hoc testing for multiple comparisons.

## 3. Results

### 3.1. AAV2^QUADYF^ Enables Stable Mosaic Gene Expression in hCMEC/D3 Spheroids

**Figure 2:**
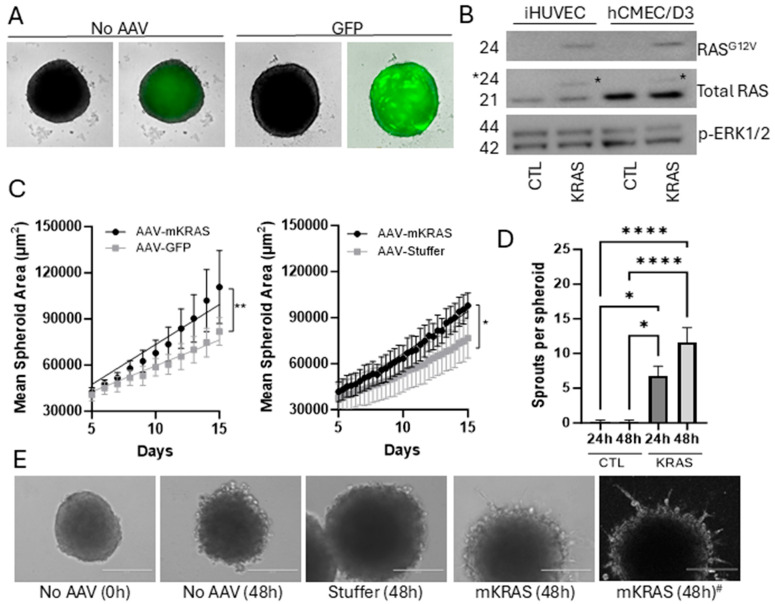
Mosaic gene expression in hCMEC/D3 cells transduced with AAV2^QUADYF^. hCMEC/D3 were seeded at a density of 1000 cells per well and after spontaneous aggregation in U-bottom wells spheroids were transduced with AAV2^QUADYF^ expressing either control genes (GFP or stuffer) or KRAS^G12V^. (**A**) Representative images of 3D spheroids showing green fluorescence (right images) related to autofluorescence (no AAV) or to mosaic GFP expression (green, right) merged with brightfield images (Incucyte SX5). Uncropped images with scale bars can be found in Supplementary Figure S1. (**B**) Western blots of total protein extracted from immortalized HUVEC-TERT2 (20 µg) or hCMEC/D3 (40 µg). Membranes were probed with a RAS^G12V^-specific antibody, an antibody targeting total RAS, and an antibody targeting phosphorylated ERK1/2. Asterisk shows the shift in band size for the recombinant KRAS protein. (**C**) Changes in spheroid size due to mutant KRAS expression were compared to GFP (left) and stuffer (right) controls using mask creation after live cell imaging in the Incucyte SX5. Data represent mean ± SEM of 3–4 independent (biological) replicates (with 6 technical replicates per plate). Simple linear regression was used to compare slopes, * *p* < 0.05, ** *p* < 0.01. (**D**) Spheroids were embedded in Geltrex growth factor-reduced basement membrane matrix and sprout formation assessed at 24 and 48 h. Data represent mean ± SEM of 3 independent (biological) replicates (with 5 technical replicates per plate). Data were analyzed using one-way ANOVA with Tukey’s post hoc analysis, * *p* < 0.05, **** *p* < 0.0001. (**E**) Representative phase contrast images showing sprout formation from embedded mutant KRAS (mKRAS) spheroids. Images were captured with an EVOS FL Inverted microscope before processing with the “Find Edges” function in Image J to improve sprout enumeration. Right image ^#^: a representative image converted with the Find Edges function. Scale bar = 200 µm.

Immortalized hCMEC/D3 were seeded in repellent 96-well U-bottom plates and AAV particles added 3 days after seeding. Spheroid formation and growth were monitored by live-cell imaging as previously established [15]. Under these conditions, we demonstrated that GFP expression could be maintained in a mosaic pattern for over 30 days without media exchange or growth factor/serum replenishment. We were unable to accurately determine the infection efficiency in the 3D model due to the mosaic pattern of expression, but previously demonstrated that this AAV2 construct could provide on average a 30% infection rate in 2D cultures [15]. Spheroids without GFP displayed weak autofluorescence, most likely representing lipofuscin accumulation and the eventual development of an acellular, necrotic core common to endothelial spheroids (Figure 2A; Supplementary Figure S1) [14,15].

Expression of mutant KRAS was established by Western blotting of total protein extracts from 2D cultures of hCMEC/D3 cells, with immortalized HUVEC-TERT2 used as a control (Figure 2B; see also Supplementary Figure S2). An antibody specifically targeting RAS^G12V^ demonstrated that expression of the mutated protein was only present in transduced cells. An antibody targeting total RAS demonstrated that mutant KRAS expression was relatively low and did not affect total RAS expression levels (densitometric ratios of RAS^G12V^ to total RAS were 0.11 +/− 0.05 for hCMEC/D3 cells and 0.28 ± 0.07 for HUVEC-TERT2 cells containing AAV-mKRAS). The mutant KRAS protein band could be distinguished from the wild-type protein by a small change in molecular weight.

No significant changes in the protein levels of phosphorylated ERK1/2 were observed under these 2D culture conditions (Figure 2B). While this potentially indicated that the KRAS protein produced was not functional, it was more likely that the combination of low mutant KRAS expression and high basal p-ERK1/2 levels (from the presence of serum/growth factors in the culture medium) limited the ability to assess small changes in down-stream signaling pathways in this 2D format. As 2D endothelial cultures undergo apoptosis in response to serum withdrawal, in contrast to 3D cultures which demonstrate increased survival characteristics, this model was used to assess the functionality of this KRAS construct in the absence of media and growth factor replenishment [14]. It was assumed that under these conditions nutrient consumption would be relatively slow relative to a 2D culture given the small number of cells within the spheroid. In control spheroids, cell viability and spheroid integrity was not compromised with extended culture, although the necrotic core expanded as expected with increasing spheroid size [14].

### 3.2. KRAS^G12V^ Increases Spheroid Size in the Early Culture Period

hCMEC/D3 spheroids transduced with AAV-mKRAS or each of the controls (AAV-GFP or AAV-Stuffer) were monitored for size and morphology for 14 days by live-cell imaging system. There was no replenishment of growth medium (bFGF or serum) during this period to ensure any small effects derived from activating KRAS expression were detectable without stimulation from exogenous growth factors. Over time, all spheroids showed a steady upward growth trajectory from day 5 post-transduction (Figure 2C). In the presence of mutant KRAS, a moderate but significant increase in growth rate was observed relative to each control. Both control constructs (AAV-GFP, AAV-Stuffer) were hence used interchangeably as negative controls.

### 3.3. KRAS^G12V^ Stimulates Spheroid Sprouting in a Reduced Growth Factor Environment

To assess angiogenic sprouting, spheroids were transferred 2 weeks after transduction into Geltrex, a basement membrane matrix with reduced growth factors. No additional media or growth factors were added. In the presence of activating KRAS, sprouts formed readily in the 24–48 h period following matrix embedding, while limited sprouting was observed in control spheroids (Figure 2D,E). Sprouts formed dynamically, both arising and receding over the observation period. Sprouts were not always uniformly expressed around the circumference and could be elongated into long projections, or short and branched. Activating KRAS^G12V^ has previously been shown to induce sprouting angiogenesis when expressed at high levels in HUVEC in both 2D and 3D formats [3]. This finding demonstrates that our construct produces a functional product demonstrating established KRAS-associated phenotypes.

### 3.4. KRAS^G12V^ Alters Spheroid Morphology After Extended Culture

**Figure 3:**
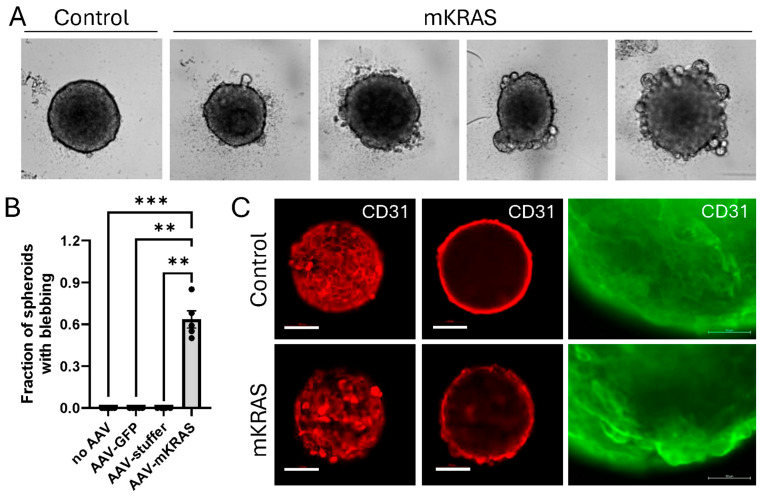
3D spheroid morphology is altered in response to mutant KRAS expression. (**A**) Representative images of control and mutant KRAS-expressing spheroids demonstrating bleb formation during extended culture (brightfield, Incucyte SX5). Uncropped images with scale bars can be found in Supplementary Figure S1. (**B**) Fraction of spheroids demonstrating blebbing were assessed at day 14. Data are representative of the mean ± SEM of at least 4 independent experiments (6 technical replicates per plate). Data were analyzed using Kruskal–Wallis non-parametric analysis with Dunn’s correction for multiple comparisons. ** *p* < 0.01, *** *p* < 0.001. Scale bar = 100 µm. (**C**) Representative images of CD31/PECAM1 immunostaining (Alexa Fluor 647, red) of control and mutant KRAS spheroids: most forward projection (left, confocal, 0 µm); circumference (center, confocal, 50 µm). Scale bar = 100 µm. Right: Enlarged image of CD31-stained blebs (Alexa Fluor 488 (green), EVOS FL Inverted microscope). Scale bar = 50 µm.

After extended culture and visual monitoring of spheroids in the live-cell imaging system it was observed that mutant KRAS caused a morphological change. The presence of mutant KRAS reproducibly induced the formation of large blebs at the spheroid surface 10 to 14 days after transduction (Figure 3A; Supplementary Figure S1). On average, 60% of replicate mutant KRAS spheroids displayed this phenotype by day 14 (Figure 3B), with the fraction of spheroids and extent of blebbing increasing with time. The appearance of blebs was dynamic in nature, with blebs periodically protruding and retracting from the surface in early stages. Some large blebs appeared to burst, releasing cellular material, before resealing of the spheroid surface; other blebs appeared to escape the primary spheroid structure and exist independently (Video S1). This contrasted with control spheroids without mutant KRAS that retained a smooth surface throughout the culture period (Video S2).

### 3.5. KRAS^G12V^-Associated Blebs Disrupt Cell-to-Cell Adhesion

**Figure 4:**
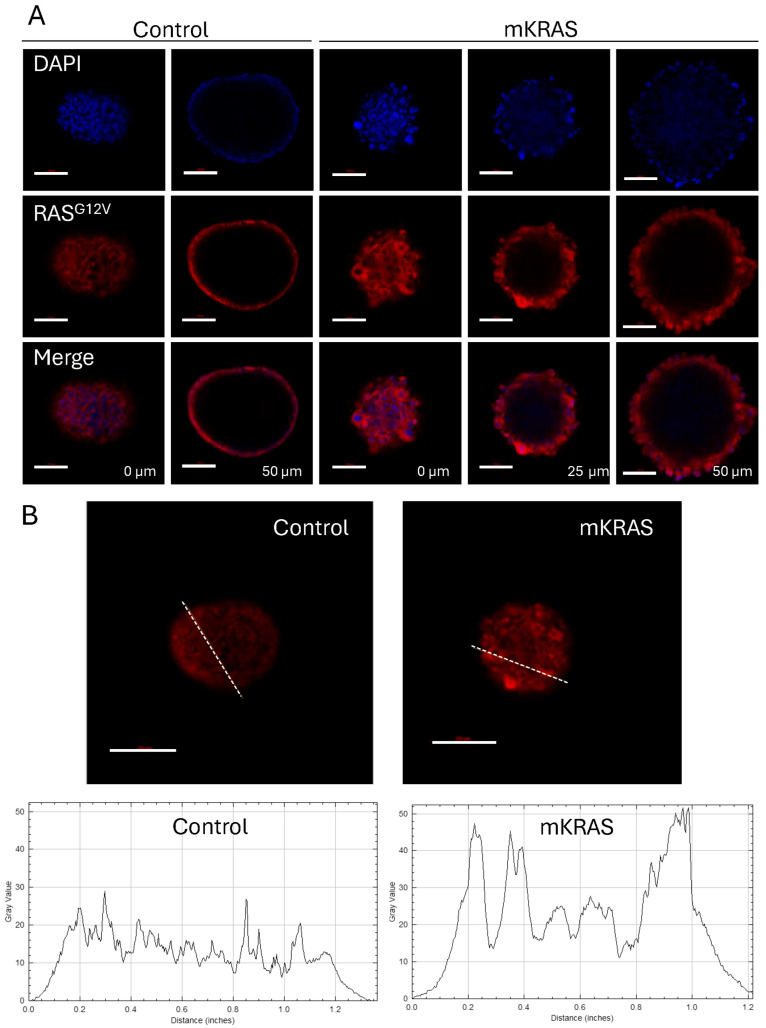
KRAS^G12V^ expression is high in hypertrophic blebbing cells at the spheroid surface. (**A**) Representative confocal images of control and mutant spheroids immuno-stained with a RAS^G12V^-specific antibody (red) showing forward and central projections. Nuclei are stained with DAPI (blue). Scale bar = 100 µm. (**B**) Representative confocal images of control and mutant KRAS spheroids immuno-stained with the RAS^G12V^-specific antibody (red) with Image J analysis of spheroid cross-sections showing increased levels of fluorescence intensity associated with hypertrophic blebbing cells at the spheroid surface. Graphs demonstrate fluorescence intensity (Gray value) versus distance (inches) as traced along the dashed lines in the corresponding images.

Immunostaining of control spheroids with CD31 demonstrated a characteristic flat, cobblestone arrangement of cells at the surface which reflects that traditionally found in 2D monolayers. CD31 (otherwise known as PECAM-1; platelet/endothelial cell adhesion molecule-1) is a junctional protein of the immunoglobulin gene superfamily involved in endothelial cell–cell adhesion [21]. In the presence of mutant KRAS, the blebbing cells retained CD31 expression but demonstrated the disruption of the cell-to-cell contacts characteristic of the surface monolayer, instead demonstrating cellular projection at the spheroid surface (Figure 3C; Supplementary Figure S3). This pattern of cell–cell disruption and cellular projection was similarly demonstrated with an antibody commercially described to target RAS^G12V^ mutant proteins. While AAV-infected controls showed a low level of background fluorescence, larger blebs at the surface of mutant KRAS-expressing spheroids showed higher intensity staining compared to surrounding cells suggesting significant mutant KRAS expression in these hypertrophic cells (Figure 4A,B).

### 3.6. KRAS^G12V^ Leads to Cellular Hypertrophy and Amoeboid Migration

**Figure 5:**
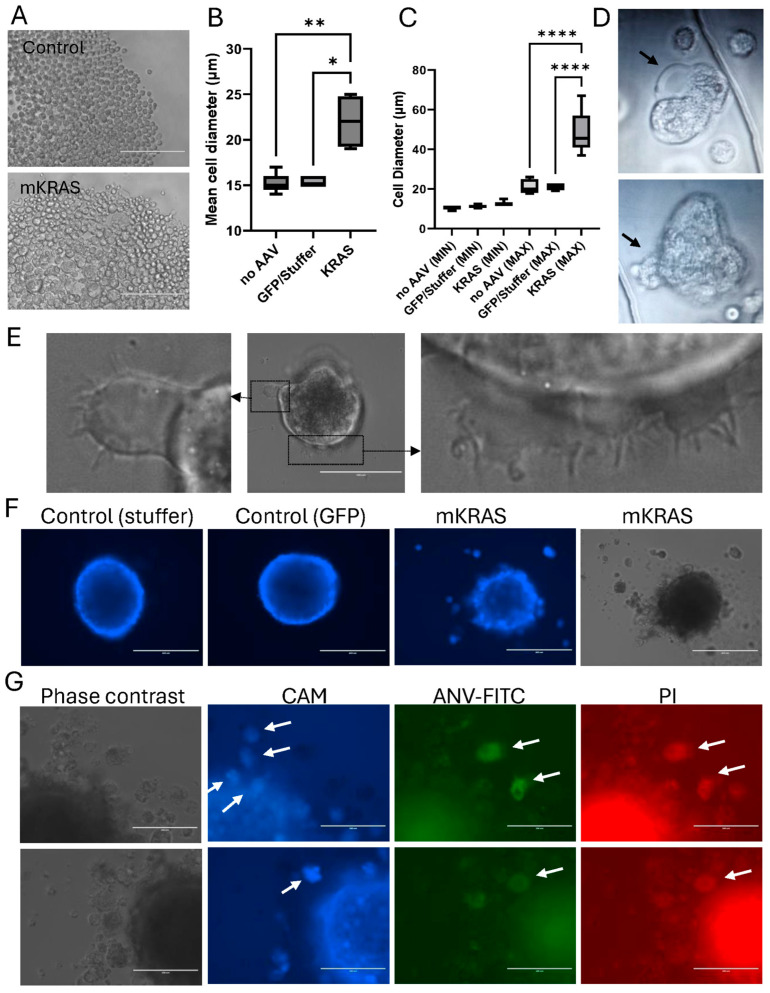
Blebbing is associated with hypertrophy and amoeboid migration. (**A**) Representative images of spheroids dissociated with trypsin-EDTA in Nunclon Sphera plates three weeks after AAV transduction (EVOS FL microscope, Scale bar = 200 µm. (**B**) Cell diameters (Kruskal–Wallis with Dunn’s post hoc) and (**C**) range of cell sizes (One-way ANOVA with Tukey’s post hoc) were determined using Image J and are shown as mean ± SEM (3 independent experiments). * *p* < 0.05, ** *p* < 0.01,**** *p* < 0.0001. (**D**) Representative images of plasma membrane blebs formed by the round-ed, hypertrophic cells released from blebbing spheroids on non-adherent plates. Images were cropped from the original video recordings (Supplementary Video S3). Black arrows highlight blebbing regions. (**E**) Representative images of ruffles, lamellipodia and filopodia formed by hypertrophic cells after transfer to adherent tissue culture plates (EVOS FL microscope); scale bar = 100 µm (central image). (**F**) Representative images showing Calcein AM uptake (blue) in control and mutant spheroids, including released blebs (EVOS FL, scale bar = 400 µm). (**G**) Representative, high magnification images of spheroids and blebs stained with Calcein AM (CAM, blue); Annexin V-FITC (ANV-FITC, green); and propidium iodide (PI; red) (EVOS FL, scale bar = 200 µm). White arrows highlight positively stained blebs after release. Note that there is limited background fluorescence in these escaped cells: those that are viable and positive for uptake of the Calcein AM Blue do not take up the PI or bind ANV-FITC, and similarly, PI+ and ANV-FITC+ cells do not retain the Calcein AM (blue) dye.

To confirm that the escaping blebs were individual cells, and not small multicellular buds, intact spheroids were digested with trypsin to assess individual cell sizes. A proportion of the cells released from mutant KRAS-expressing spheroids appeared hypertrophic (Figure 5A). Mean cell diameters and associated size ranges were greater in spheroids expressing KRAS^G12V^ compared to any of the negative controls, where cell sizes remained highly uniform (Figure 5B,C).

Immediately after trypsin digestion, the large hypertrophic cells released from the mutant spheroids displayed characteristics of highly motile amoeboid-like cells on the non-adherent plates (Figure 5D; Video S3). These large, rounded cells were highly deformable, themselves demonstrating large, dynamic membrane ruffles and bleb-like protrusions of the plasma membrane. The cellular blebbing was rapid, with multiple blebs forming and retracting at the plasma membrane within the space of a few minutes. Rapid blebbing is a hallmark of amoeboid transition [22]. Upon transfer of these large cells to an adherent surface, the cells remained rounded and non-adherent but decreased bleb formation and increased lamellipodia and filopodia production (Figure 5E).

Blebs at the spheroid surface were mostly viable, staining positively for Calcein AM uptake (Figure 5F). However, blebs that had escaped the spheroid surface eventually became negative for Calcein AM and positive for the apoptotic markers, ANV-FITC and PI (Figure 5F,G). Cell blebbing was initially described as a feature of apoptosis, however in apoptotic cells, blebs are not retractable [21]. This contrasts with the dynamic bleb formation observed in this study. While the blebbing cells in our study eventually became apoptotic, this occurred only after extended detachment from the primary spheroid, consistent with the known anchorage-dependence of endothelial cells and induction of anoikis in the absence of cell-to-cell or cell-to-matrix adhesion [23].

### 3.7. Blebbing Can Be Reversed with MEK, mTOR, and Rho/ROCK Inhibitors

**Figure 6:**
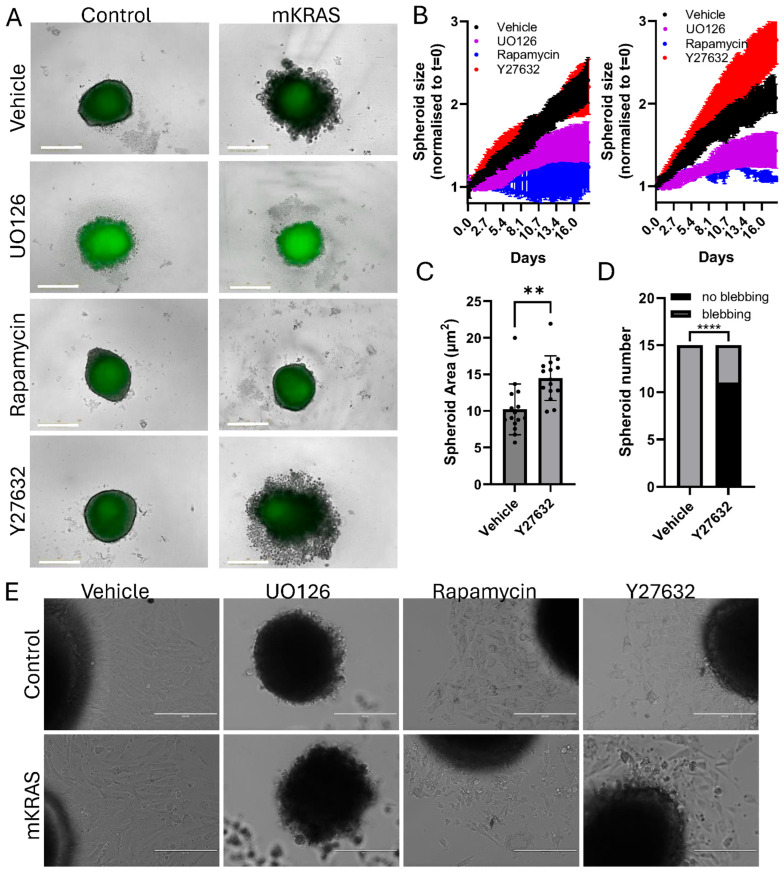
Phenotype reversal in the presence of MEK, mTOR and Rho/ROCK pathway inhibitors. (**A**) Representative brightfield and fluorescent images of control (left) or mutant (right) spheroids 18 days after addition of vehicle (DMSO, 0.1%), MEK inhibitor (UO126, 10 µM), mTOR inhibitor (Rapamycin, 10 µM), or Rho/ROCK inhibitor (Y27632, 10 µM). (Incucyte SX5, scale bar = 400 µm). (**B**) Representative live-imaging data of a single experiment (mean ± SD, 3–6 technical replicates per group) demonstrating typical spheroid growth curve responses over 18 days after 10 µM inhibitor addition. Left graph: control spheroids. Right graph: mutant KRAS spheroids. Spheroid size was determined by mask creation and normalized to time zero (Incucyte SX5). (**C**) The area of mutant KRAS spheroids treated with vehicle or Y27632 were independently measured using the freehand drawing tool in Image J. (mean ± SD, n = 15 spheroids per group obtained from 3 independent experiments). Data were analyzed using Student’s *t*-test (unpaired), ** *p* < 0.01. (**D**) The fraction of mutant KRAS spheroids that demonstrated blebbing after treatment with vehicle or Y27632. Data were inserted into a 2 × 2 contingency table and analyzed using Fisher’s Exact Test, **** *p* < 0.0001. (**E**) After 18 days of inhibitor treatment spheroids were transferred to adhesive flat-bottom tissue culture plates to assess outgrowth (EVOS FL, scale bar = 200 µm; higher magnification images can be found at Supplementary Figure S5).

MEK and mTOR pathways are known KRAS targets. A single addition of either UO126 or Rapamycin in 2-week-old spheroids rapidly stopped spheroid growth and reversed the blebbing phenotype in mutant spheroids, though growth of control spheroids was also affected (Figure 6A,B). Amoeboid migration is known to be controlled more specifically by downstream signaling through Rho/ROCK pathways which control cytoskeletal organization [24]. Treatment with a Rho/ROCK inhibitor (Y27632) stimulated a moderate increase in spheroid size and a reduction in cell hypertrophy and blebbing in mutant KRAS spheroids consistent with an amoeboid state (Figure 6A–D; Supplementary Figure S4). Additionally, no significant change was observed in control spheroids treated with Y27632 (Figure 6A,B).

MEK inhibition increased spheroid autofluorescence (lipofuscin) over time, suggesting these cells were experiencing increased stress. Upon transfer of these spheroids to a flat-bottom, adhesive tissue culture plate the UO126-treated spheroids disintegrated, leaving an acellular core, unable to produce any cellular outgrowth (Figure 6E). In contrast, Rapamycin and Y27632-treated spheroids were transferable; however, the extent of outgrowth was limited in comparison to vehicle-treated spheroids and the cells displayed abnormal morphologies (Figure 6E; higher magnification images in Supplementary Figure S5). In the presence of mutant KRAS, morphologies consistent with cellular senescence (large, flat, multi-nucleated) were more prevalent in adherent cells [19]. The large blebbing cells retained their rounded morphology and non-adherent properties.

## 4. Discussion

Using a simple endothelial spheroid model this study demonstrated that in this 3D format, wild-type brain microvascular endothelial cells retained a flattened cobblestone appearance at the spheroid surface with retention of endothelial markers. Subsequent expression of activating KRAS^G12V^ mutations common to sporadic AVMs independently induced angiogenic characteristics typically associated with the endothelium of human brain AVM and with earlier KRAS studies [3], showing that we could express a functional mutant KRAS construct in this model. Uniquely, after extended culture without constant growth factor stimulation, novel morphological and phenotypic features associated with mesenchymal-amoeboid transition (MAT) and amoeboid-like migration were observed to be driven by these constitutive KRAS mutations and absent in control spheroids.

MAT-driven blebbing has rarely been observed in an endothelial context but has been commonly described in tumor spheroids [25,26,27]. Multiple studies have identified stem-like characteristics in AVM endothelial cells associated with endothelial–mesenchymal transition (EndoMT) [28]; however, no previous studies have demonstrated associations with MAT. MAT represents an additional step toward phenotypic immaturity that forms part of the cancer cell continuum [22]. MAT is characterized by the formation of rounded, highly deformable cells that move via the rapid (seconds to minutes) protrusion and contraction of plasma membrane blebs, a hallmark of this cellular state [22,24]. Cellular blebbing is thought to occur through localized decoupling of the cytoskeleton from the plasma membrane and relies on weak cell–matrix and cell–cell adhesion; proteolytic degradation is not required as deformable cells squeeze through existing spaces in the matrix [29]. In contrast, EndoMT is characterized by the formation of elongated, spindle-like cells with migration directed from a leading edge and chemokine-stimulated lamellipodia and filopodia formation. Movement is dependent upon strong integrin attachment as well as matrix metalloprotease (MMP) release, to allow tunneling through the matrix [29].

EndoMT and MAT states are interchangeable, reflecting responses to local stimuli. Cues for control of amoeboid migration in cancer cells include physical confinement and nuclear deformation; non-adherent surfaces can also increase MAT conversion [30]. In endothelial cells, MAT has rarely been observed, however Chillà et al. demonstrated that broad MMP inhibition can stimulate amoeboid angiogenesis in both mature and progenitor endothelial cells [31,32]. Protease inhibition appears to prevent MMP-dependent mesenchymal migration and in doing so can stimulate the alternate amoeboid-migration pathway when conditions allow. It is possible that confinement of cells in the 3D model interacted with the phenotypic plasticity of mutant KRAS-expressing cells in this study to induce amoeboid migration and may be an in vitro artifact but demonstrates that the highly plastic nature of the KRAS mutation in endothelial cells could produce unforeseen phenotypes under the right conditions. While 3D spheroid models provide added physiological relevance not attainable in 2D cultures such as increased maturity and survival characteristics [14], endothelial cells do not typically live in clustered arrangements like tumor cells, but in 2D monolayers at the luminal vascular surface. However, multilayered structures are evident in the abnormal AVM endothelium [33] and hypertrophic endothelial cells have been observed in the arterialized veins of human brain AVMs [34]. We cannot yet exclude that this unique vascular environment may contribute to acquisition of this phenotype in the presence of this highly plastic mutation during pathophysiological development [33]. Unfortunately, distinct molecular markers for this amoeboid transition are currently lacking and characterization is often defined by phenotypic traits and molecular signaling pathways as described here, rather than via analysis of single surface markers which may vary between cell types [22,24]. Ongoing RNAseq and proteomic analysis of this 3D model with and without mutant KRAS expression may assist with the identification of novel MAT markers for future testing with ex vivo human AVM tissue.

In tumor cells, a key driver of amoeboid migration that acts downstream of RAS/MEK/ERK is RhoA, as a small GTPase that activates ROCK (Rho-associated protein kinase) [24,26]. Other small GTPases from the Rho family, Rac1 and Cdc42, are important in mesenchymal migration and the formation of lamellipodia and filopodia, respectively, through actin polymerization [26]. RhoA/ROCK activity leads to the phosphorylation of myosin II and actin-myosin contractility. Localized myosin II contraction pulls the cytoskeleton away from the plasma membrane, allowing cytosolic contents to temporarily swell these regions of the cell. Eventually the cytoskeletal elements reconnect causing retraction of the bleb, propelling the cell forward [26]. The ability of the RhoA/ROCK inhibitor to decrease the formation of hypertrophic, blebbing cells in this study provided further evidence that these mutant cells exhibit an amoeboid state [24]. Y27632 demonstrated a very specific effect on bleb formation in the presence of mutant KRAS expression without significant effects on the growth of either control or mutant cells.

Inhibition of upstream pathways with UO126 and Rapamycin also reversed bleb formation but concomitantly demonstrated significant effects on spheroid growth in both controls and mutant cells. The ability of the latter drugs to regress KRAS-associated phenotypes in this model is consistent with preliminary studies in humans showing that drugs targeting these pathways can in part reduce volume and ameliorate symptoms in AVM patients, though these studies have been predominantly on extra-cranial, low-flow lesions [35,36,37,38]. The toxicity of the MEK inhibitor observed in this study after extended culture is consistent with the potential for off-target effects observed in these human studies. Rapamycin and Y27632 showed less toxicity, however morphological changes that reflected senescence-like phenotypes after inhibitor removal and outgrowth were observed, particularly in the mutant KRAS background. Further studies are required to monitor this aspect of cell recovery or cell stasis over the longer term and with a wider dose range but may provide important information regarding the mechanisms by which these drugs affect the mutated endothelium. While the overt toxicity of UO126 may have been caused by the extended period of inhibition (2 weeks) used in this study, the live-cell imaging system allowed us to take an exploratory approach not typically available with short-term 2D cultures. For high-throughput applications, future experiments could certainly employ shorter periods of inhibitor treatment.

Whether Rho/ROCK inhibitors have any potential in ameliorating KRAS-driven AVM angiogenesis in humans will depend on obtaining further in vivo evidence that confirms at least a partial role for MAT conversion in the endothelial cells of sporadic AVMs. The cellular plasticity induced by this mutation may also reveal a role for combination treatments involving this pathway. Combined treatments with Rac1 (mesenchymal) and RhoA (amoeboid) inhibitors may be necessary to block both mesenchymal and amoeboid migration pathways in angiogenesis as it has been shown that inhibition of just one of these pathways can lead to a stimulatory effect on the other. For example, Chilla et al. induced MAT in mature endothelial cells by inhibiting mesenchymal migration with a broad protease inhibitor [32]. Alternatively, Rho/ROCK inhibitors could be used in combination with anti-vascular endothelial growth factor (VEGF) agents such as Bevacizumab to inhibit both migration pathways. Chilla et al. demonstrated that anti-VEGF agents could inhibit angiogenesis associated with mesenchymal migration but not amoeboid migration and suggested that the limited efficacy and resistance of single anti-VEGF agents in cancer therapy may in part be due to the drug-associated induction of MAT [31,32]. Similarly, AVMs are known to exhibit high levels of VEGF in the dysfunctional endothelium [39,40] yet have shown relative resistance to Bevacizumab in studies to date [41].

This model and live imaging approach could easily be adapted to a 364-well plate format for high-throughput testing of drug libraries for potential drug repurposing. Future adaptation of this model to express other AVM-associated mutations such as KRAS^G12D^ and activating BRAF can be easily carried out with novel AAV constructs. While these mutations activate the same upstream MEK/ERK pathway, studies in cancer cells have shown subtle but important differences in signaling activity between these mutation types, that may contribute to variable traits in AVM patients or affect pharmaceutical susceptibility [42,43]. It would be interesting to determine specifically whether the acquisition of an amoeboid phenotype is also possible in the presence of these alternative activating mutations.

There were several limitations to this study. Firstly, the introduction of AAV constructs after spheroid formation reduced the reliability of blebbing induction. When establishing this approach in our earlier study we investigated AAV transduction both pre- and post-spheroid formation [15]. We found by transducing cells in 2D then trypsinizing and seeding for spheroid formation, we obtained spheroids with a very even distribution of GFP throughout the structure. However, by transducing established spheroids with AAV we achieved a more random, mosaic distribution at the surface which we considered to be more representative of the patterns found in brain AVMs. Our studies hence continued with the latter approach and while being highly informative during this discovery period, it meant that more replicates were required in each experiment to ensure statistical significance. Future high-throughput studies might consider using the former approach to improve reproducibility in KRAS expression and reduce replicate requirements. Secondly, while the ability to simultaneously assess spheroid size and visualize morphological characteristics such as blebbing in the live-cell imaging system was a significant advantage that allowed rapid qualitative assessment of cellular phenotype and first-pass analysis of drug responses that affected spheroid size, we did not assess a more quantitative method for spheroid bleb formation at this stage, but further development or identification of Image J plug-ins or macros would be valuable for future high-throughput applications.

## 5. Conclusions

This is the first study to examine mutant KRAS expression in a spheroid model using brain endothelial cells, with validity of the model evident through consistency with other short-term experiments performed with mutant KRAS in HUVEC-derived 2D or 3D cultures. The ability to extend the period of spheroid culture without the need for media replenishment in this model identified a unique migratory pathway inducible in KRAS-mutated endothelial cells and highlights the extreme cellular plasticity induced by this mutation. Our studies highlight that 3D endothelial spheroids can provide novel information on KRAS-associated phenotypes, but further studies are required to establish the in vivo relevance of MAT transitions to human AVM development, clinical outcomes (rupture risk), or drug susceptibility.

## Data Availability

All data generated or analyzed in this study are included in this article or in the Supplementary section. The original data are available upon reasonable request. Further enquiries can be directed to the corresponding author.

## Supplementary Materials

The following supporting information can be downloaded at: https://www.mdpi.com/article/10.3390/cells15010022/s1, Figure S1: Incucyte SX5 imaging of 3D endothelial spheroids; Figure S2: Western blots; Figure S3: Expression of CD31 on endothelial spheroids; Figure S4: Representative spheroids treated with Rho/ROCK inhibitor. Figure S5: Cellular outgrowth from treated spheroids; Video S1: Blebbing of KRAS-mutant spheroids; Video S2: Growth of wild-type endothelial spheroids; Video S3: Amoeboid-like behavior of KRAS-mutant cells.

## Abbreviations

The following abbreviations are used in this manuscript:

AAV	Adeno-associated virus
AVM	Arteriovenous malformation
ANV-FITC	Annexin V-fluorescein isothiocynate
EndoMT	Endothelial–mesenchymal transition
GFP	Green fluorescent protein
hCMEC	Human cerebral microvascular endothelial cells
HUVEC	Human umbilical vein endothelial cell
KRAS	Kirsten rat sarcoma viral oncogene homolog
MAT	Mesenchymal-amoeboid transition
