# Supplementary material for: Activating KRAS Mutations Expressed in 3D Endothelial Spheroids Induce Blebbing Morphologies Associated with Amoeboid-like Migration
Source: Cells. 2025 Dec 22;15(1):22. doi: 10.3390/cells15010022 (PMC12784968; doi:10.3390/cells15010022)
Supplement: Supplementary file 1 [file cells-15-00022-s001.zip › cells-4020381-resubmit-Supplementary material_CELLS_REVISED.pdf]

Supplementary Materials

Activating KRAS mutations expressed in 3D endothelial spheroids induce blebbing morphologies associated with amoeboid-like migration

Lucinda S McRobb[1], Vivienne S Lee[1], Marcus A Stoodley[1]

[1] Macquarie Medical School, Faculty of Medicine, Health and Human Sciences, Macquarie University, Sydney, New South Wales, 2109, Australia

Corresponding author

Dr Lucinda S. McRobb (PhD)

Lucinda.mcrobb@mq.edu.au

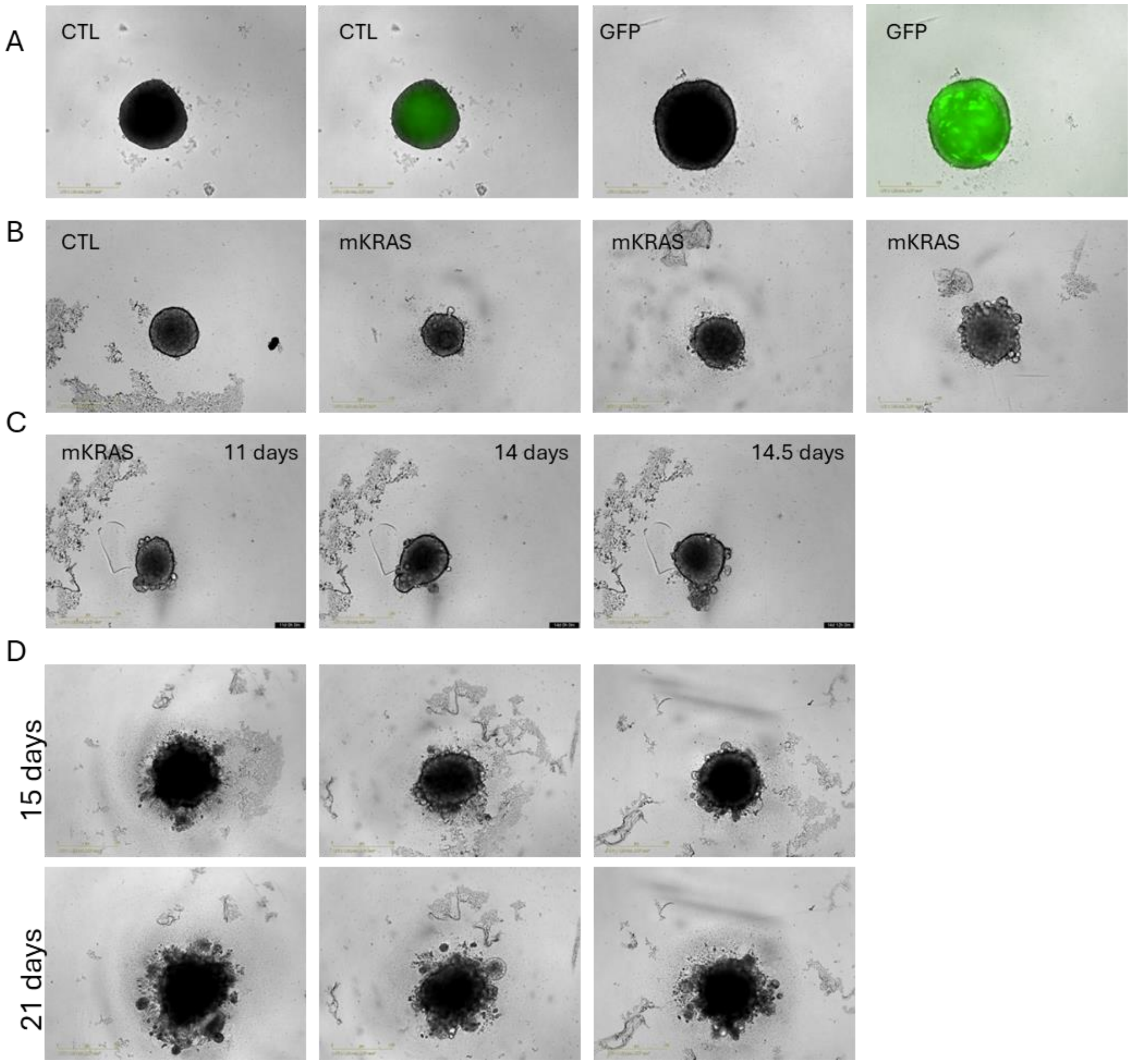

Figure S1: Incucyte SX5 imaging of 3D endothelial spheroids. (A) Representative brightfield and fluorescent images (full size, uncropped) of non-infected and AAV-GFP transduced spheroids after 30 days of incubation. Autofluorescence (green) is observed in most control spheroids and increases steadily with time of incubation. GFP expression (green) is mosaic. (B) Representative images of control (stuffer) and mutant KRAS^{G12V} transduced spheroids after 10-14 days showing variable levels of surface blebbing. (C) Representative images of single spheroid showing large bleb that appeared to burst after release from the spheroid. Images shown were captured at day 11, day 14 and day 14.5. (D) Representative images of 3 mutant KRAS spheroids showing large blebs escaping the primary spheroid over time. All images were captured using an Incucyte SX5 (Sartorius) live-cell imaging instrument. Scale bar (lower left corner) = 400 µm on all images.

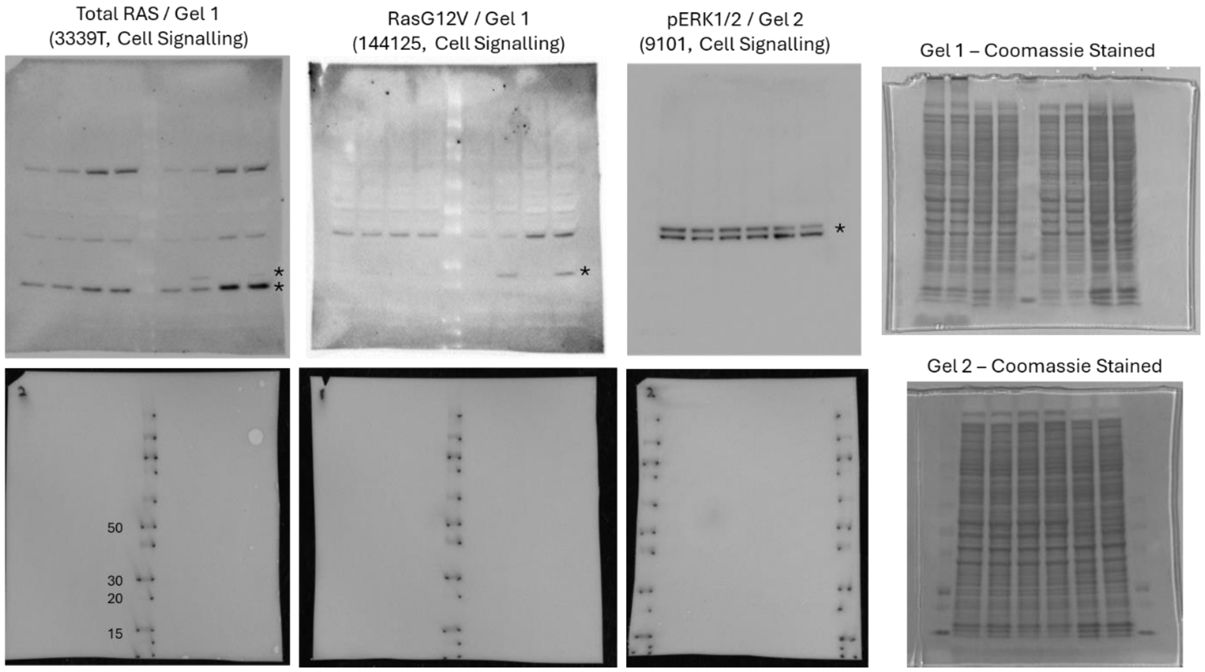

Figure S2: Western blots. Original uncropped images of western blots (enhanced chemiluminescence, top row) and associated white light images of PVDF membranes demonstrating size ladders (bottom row). Molecular weight bands of 15, 20, 30 and 50 kDa are highlighted. Asterisk shows relevant bands. Right: Coomassie-stained polyacrylamide gels are shown for each blot to demonstrate equivalent sample loading between gel lanes.

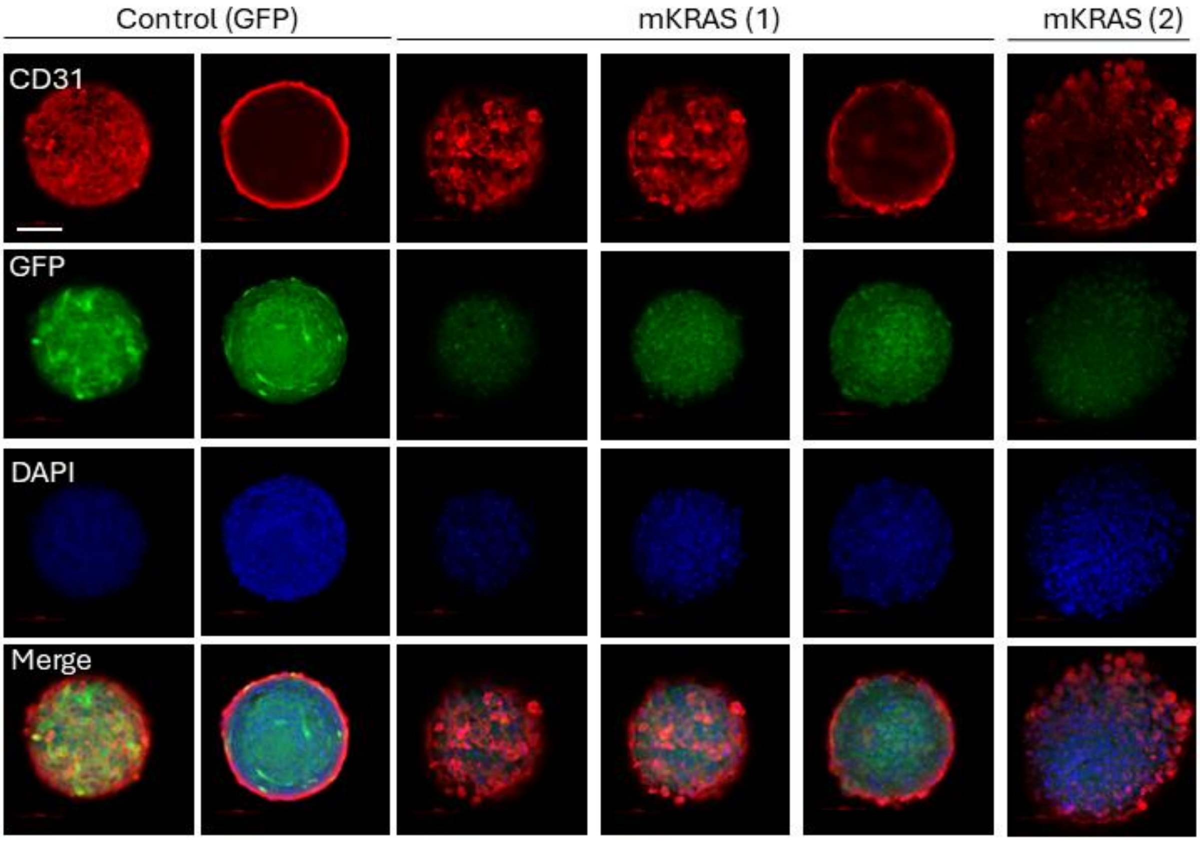

Figure S3: Expression of CD31 on endothelial spheroids. hCMEC/D3 spheroids were transduced with AAV-GFP or AAV-mKRAS and after 3 weeks were immunostained for the endothelial marker, CD31 (AF647, red). RIGHT: Representative confocal images of a control spheroid demonstrating mosaic expression of GFP (green) and a cobblestone pattern of CD31 expression (red) at forward and medial projections. CENTRE and RIGHT: Spheroids expressing mutant KRAS (mKRAS) demonstrated moderate to extensive blebbing at the surface which disrupted the cobblestone pattern of CD31 staining. mKRAS (1) demonstrates three different confocal projections (0 - 50 µm) through one representative spheroid with autofluorescence (green) observed in the spheroid core. mKRAS (2) shows a second representative spheroid with more extensive blebbing. DAPI is use for nuclear staining (blue). Scale bar = 100 µm.

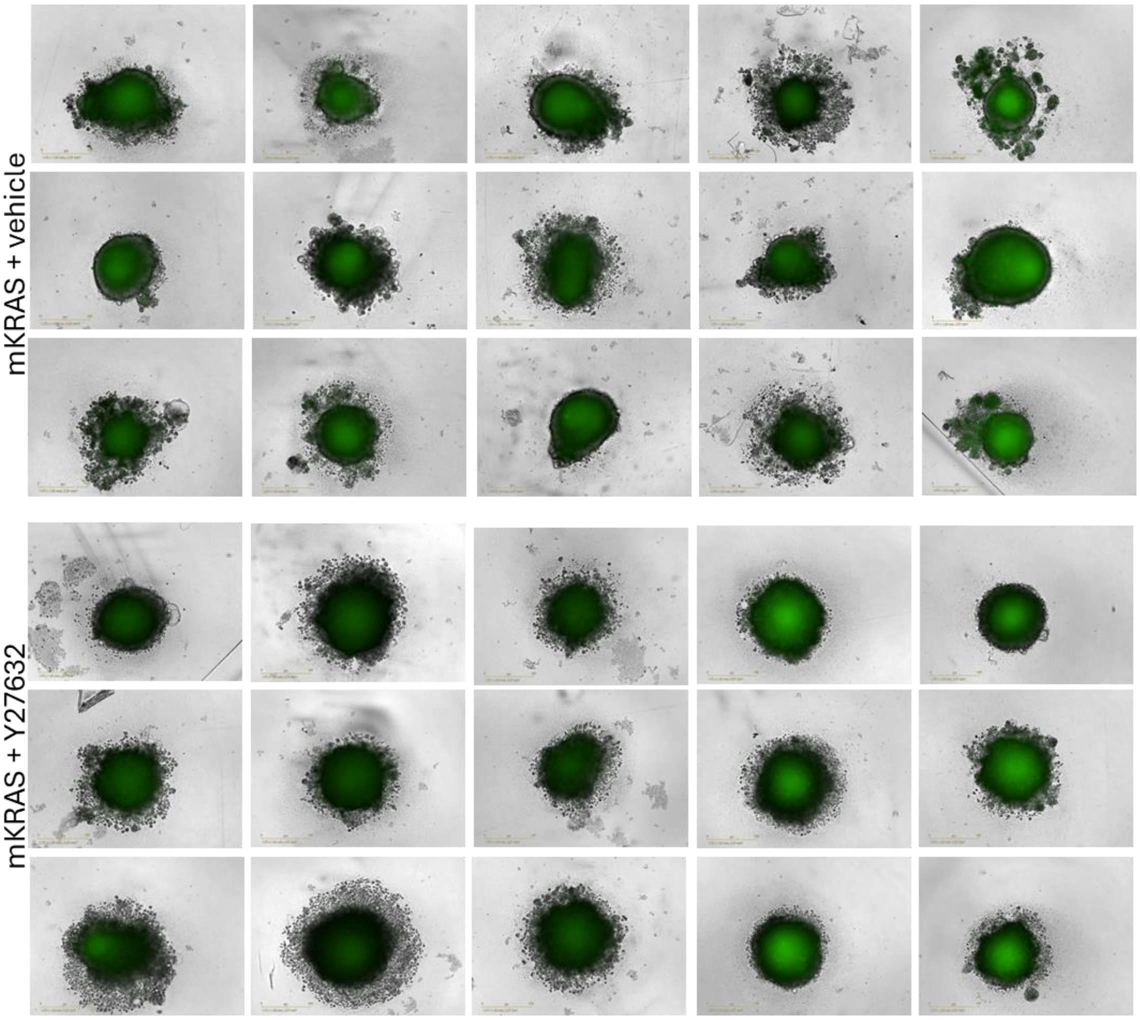

Figure S4: Representative spheroids treated with Rho/ROCK inhibitor. Spheroids were incubated with AAV-mKRAS for 2 weeks before addition of the Rho/ROCK inhibitor (10 µm) or DMSO (0.1%, vehicle). Images (brightfield with autofluorescence) of 15 spheroids acquired across 3 independent experiments were captured by live-cell imaging with an Incucyte SX5 Instrument. Y27632 (lower images) did not inhibit spheroid growth but reduced the formation of KRAS-dependent cellular hypertrophy and surface blebbing. Scale bar (lower left corner) = 400 µm on all images.

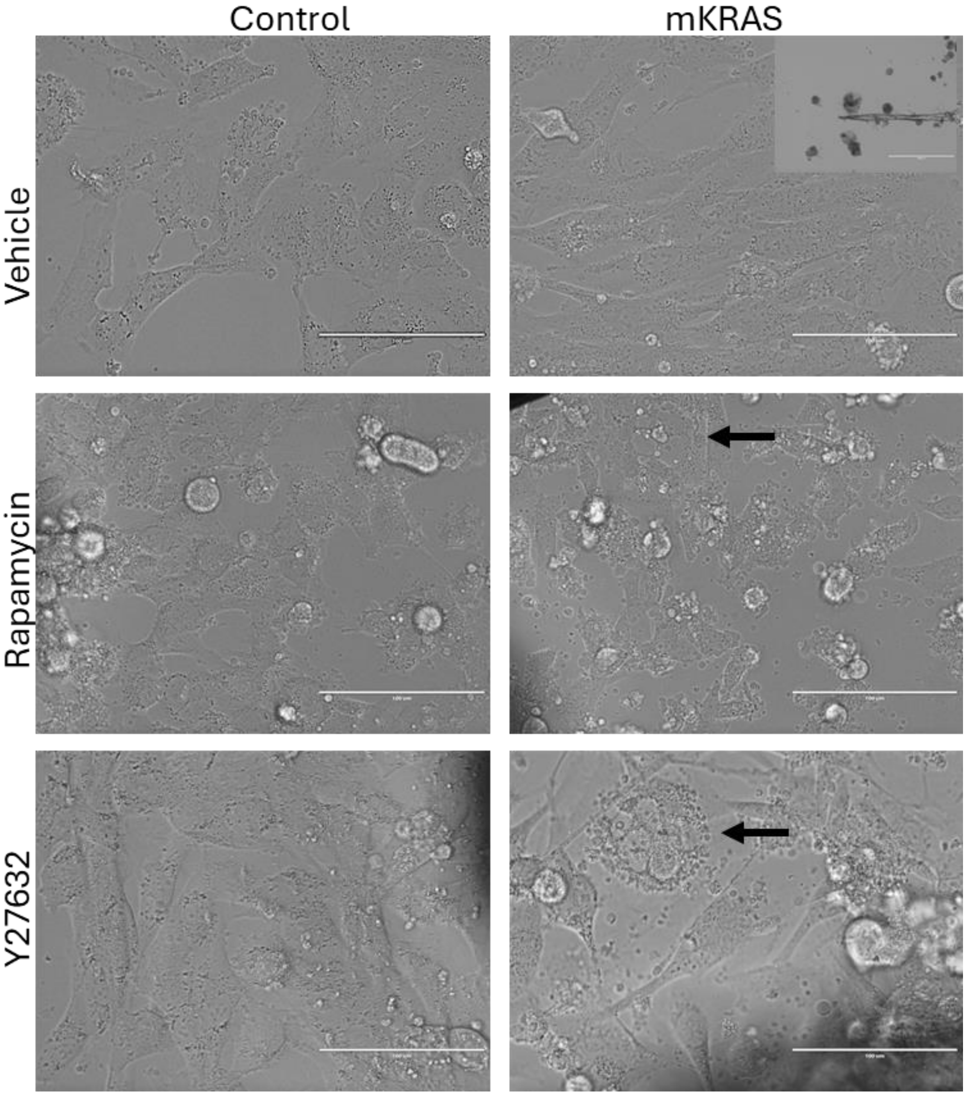

Figure S5: Cellular outgrowth from treated spheroids. After 18 days of treatment with mTOR (rapamycin) or Rho/ROCK (Y27632) inhibitors, spheroids transduced with control (stuffer) or mutant KRAS were transferred to standard 24-well tissue culture plates containing 0.5 mL of fresh EGM2 growth media and cellular outgrowth monitored for 1-2 days. Images of adherent cells were captured using an EVOS FL microscope. Inset shows large blebs from the mKRAS spheroids that retained their rounded shape and loss of adhesive capabilities. Large multinucleated senescent-like cells were observed predominantly in cells with mutant KRAS expression after mTOR or Rho/ROCK inhibition (arrows). MEK inhibition (with UO126) led to complete toxicity after 18 days and no cellular outgrowth (not shown). Scale bar = 100 µm (large images); scale bar = 400 µm (inset).
